# In vitro evaluation of anti-proliferative, anti-inflammatory and pro-apoptotic activities of the methanolic extracts of *Andrographis nallamalayana* Ellis on A375 and B16F10 melanoma cell lines

**DOI:** 10.1007/s13205-016-0529-0

**Published:** 2016-10-01

**Authors:** Guroji Purushotham, Yerukala Padma, Yusuf Nabiha, R. R. Venkata Raju

**Affiliations:** 1 Phytomedicine Division, Department of Botany, Sri Krishnadevaraya University, Anantapuram, Andhra Pradesh 515003 India; 2 Department of Dermatology and Skin Diseases Research Center, University of Alabama at Birmingham (UAB), Birmingham, AL 35294 USA

**Keywords:** *Andrographisnallamalayana* (AN), Cancer, Skin cancer, Cell cycle arrest, Apoptosis

## Abstract

Natural plant products have been widely used in controlling cancer with fewer or no side effects and the use of plant extracts as complementary to synthetic medicine is gaining increased popularity. Members of *Andrographis* plants possess important medicinal properties. In the present study, anti-cancerous properties of *Andrographis nallamalayana* (AN) were tested on A375 and B16F10 skin melanoma cancer cell lines. The leaf extracts of AN significantly reduced the cell viability and cell survival of skin cancer cell lines, achieved by MTT assay and clonogenic assays, respectively. Further, TUNEL assays revealed that AN extracts induces the apoptosis. Western blot analysis revealed that AN leaf extracts reduced the expression of Bcl-2, an anti-apoptotic protein and induced the expression of proapoptotic molecules such as Bcl-2 associated death promoter protein (BAD), Bcl-2 associated X protein (BAX) and cleaved caspase-3. Moreover, the qRT-PCR and western blot analysis demonstrated the reduced expression of G2/M phase proteins cdk1, cyclin B1 and increased expression of p53, cyclin-dependent kinase 1 inhibitor, p21. Further, immunofluorescence analysis revealed that AN reduced the NF-κB nuclear translocation, luciferase reporter assays demonstrated reporter gene activation. qRT-PCR assays showed that AN significantly reduced the expression of NF-κB target genes. The results concluded that the extracts of AN exhibited significant anti-proliferative, anti-inflammatory and pro-apoptotic activities on melanoma skin cancer cell lines.

## Introduction

Medicinal plants were being used to treat various chronic diseases from ancient times. Natural plant products have shown their potentiality in controlling cancer while exhibiting less or no side effects and the use of plant extracts as complimentary to modern medicine is gaining increased popularity. Up to 80 % population in the developing countries is depending on herbal medicine to heal different diseases and they are inaccessible to allopathic medicine (Ekor 2014; Pandey et al. 2013; Mzid et al. 2012; Rates 2001). Some species of *Andrographis* gained its own importance due to their important medicinal values and an effective anti-cancerous source (Agbonlahor et al. 2014; Dhiman et al. 2012; Abhishek et al. 2010). Members of *Andrographis* are widely used in various ailments; particularly, *Andrographis paniculata* Nees (*A. paniculata*) used in the treatments of wide varieties of diseases in traditional systems of the medicine (Agbonlahor et al. 2014). *A. paniculata* extracts were tested against variety of cancer cell lines and reported that compounds isolated from the extracts exhibited potent anti-tumor activity against all the investigated cancer cell lines (Luo et al. 2014; Lin et al. 2014; Geethangili et al. 2008; Rajagopal et al. 2003). It has also been showed that the methanol extract of *A. paniculata* was subjected to fractionation and reported that dichloromethane fraction of methanolic extract possessing cytotoxic activity and also potent immunostimulating activity (Sagadevan et al. 2015; Churiyah et al. 2015; Ajay et al. 2004). However, it was also reported that there were also adverse side effects of *A. paniculata* which may include gastric upset, headache, bitter taste and fatigue. Moreover, reports revealed that the high doses of *A. paniculata* may affect the normal functions of liver (Prakash et al. 2013). Hence, exploring more species of the *Andrographis* for medicinal properties including anticancer properties may prove to be worthwhile.

AN which was used in this study is procumbent herb, endemic to Nallamala hills, Andhra Pradesh, India. This herb was known to be used for treating mouth ulcers (Venkata Ratnam and Venkata Raju 2005) as well as leukoderma in folklore medicine and recent reports showed that parts of this herb were persisting antimicrobial and anti-psoriatic properties, respectively (Padma and Venkata Raju 2013; Parlapally et al. 2015). However, the anticancerous properties of AN are hitherto unknown. In the present study, we examined the anti-cancerous properties such as antiproliferative and pro-apoptotic effects of the methanolic extracts of AN on skin cancer cell lines, A375 and B16F10 and unraveled the detailed molecular mechanism of action of the test extracts.

## Materials and methods

### Reagents

A375 and B16F10 cell lines were obtained from American Type Culture Collection (ATCC). DMEM media, antibiotics penicillin and streptomycin were purchased from Gibco BRL (CA, USA). Fetal bovine serum (FBS) was purchased from Hyclone Laboratories Ltd (Logan, UT). TNF-α, trypsin–EDTA, protease inhibitor cocktail were purchased from Sigma Chemicals (St Louis, MO, USA). All primary antibodies [BAX, p-BAD, BAD, B cell lymphoma-extralarge (Bcl-XL), p53, cyclin B1, Cell division cycle 25c (Cdc25c), p-cdc25c, caspase-3, and GAPDH] were purchased from Cell Signaling Technology (Beverly, MA, USA) and secondary antibodies from Sigma Chemicals (St Louis, MO, USA).

### Preparation of plant extract

The well-developed matured plant parts of AN were collected from the forests of Nallamalais, central region of Eastern Ghats. The specimen was identified with the help of the regional floras and the voucher specimen was deposited at Sri Krishnadevaraya University Herbarium (SKU), Ananthapuramu. The collected plant parts were washed with tap water, shade dried, powdered and extracted with petroleum ether, ethyl acetate and methanol using Soxhlet apparatus for 6 h. The extracts were filtered through Whatman number 1 filter paper and the filtrates were concentrated under reduced pressure at 40 °C using a rotoflash evaporator. Dried powder was dissolved in dimethyl sulfoxide (DMSO) for treatments.

### Cell culture

A375 and B16F10 cell lines were cultured in DMEM media supplemented with 10 % FBS, 100 IU/ml penicillin and 100 μg/ml of streptomycin in a humidified atmosphere with 5 % CO_2_ at 37 °C. Cell lines were passaged in the laboratory for less than 6 months.

### MTT cell viability assays

The effects of the extracts of AN on cell proliferation of A375 and B16F10 cell lines were performed using MTT cell viability assays. Inhibition of cell proliferation was measured by the reduction of 3-(4,5-dimethylthiazol-2-yl)-2,5-diphenyltetrazolium bromide (MTT) to formazan. A375 and B16F10 cells were seeded (2000 cells/well) in 96 wells plates, after overnight incubation cells were treated with varying concentrations of methanolic extracts of leaf and stem of AN for 72 h (10, 50, 100 and 150, 200, 250 and 300 μg) or vehicle (0.1 % DMSO). Then MTT reagent was added to the medium in each well and incubated for 4 h at 37 °C. Then, reduced formazan crystals were solubilized in DMSO was then the optical density values were measured at 540 nm on micro plate reader. All treatments were performed in triplicate and results were expressed as mean ± SE.

### Clonogenic assays

Effect of AN on colony formation ability of A375 and B16 F10 cells was performed using clonogenic survival assays. A375 and B16F10 cells plated at a density of 500 cells/well in six well plates. After overnight incubation cells were treated with AN leaf extracts or vehicle (0.1 % DMSO) for 48 h. Then cells were allowed to grow for additional 7 days and colonies were stained with 0.5 % methylene blue in 50 % methanol. Colonies that contain more than 50 cells were counted. All treatments were performed in triplicate and results expressed as mean ± SE.

### Terminal deoxynucleotidyl transferase dUTP nick end labeling (TUNEL) assay

An in situ apoptosis detection kit was used to detect DNA fragmentation in accordance with the manufacturer’s procedure (Roche, Indianapolis, IN, USA). Briefly, B16F10 and A375 cells were grown on chamber slides, treated with AN for 48 h. The cells were then fixed with 4 % paraformaldehyde for 10 min, and then incubated for 60 min with a reaction mixture containing fluorescein conjugated-dUTP and terminal deoxynucleotidyl transferase. Positively stained fluorescein-labeled cells were visualized and photographed using a fluorescence microscope. DAPI was used to visualize the nuclei.

### Western blotting

50 µg of total cellular lysates was mixed with SDS sample buffer, boiled for 5 min and subjected to electrophoresis on 10 and 12 % SDS–polyacrylamide gels. Then resolved proteins were transferred onto PVDF membranes for overnight at 4 °C. Membranes were incubated in blocking buffer [5 % non-fat dry milk in tris buffered saline (TBS) (10 mM Tris (pH 7.5), 150 mM NaCl] for 2 h, and then incubated with the primary antibodies in blocking buffer for overnight at 4 °C. The membranes were then washed with TBS-T (TBS contacting 0.1 % Tween-20) thrice for 10 min each and incubated with secondary antibody conjugated with horseradish peroxidase for 2 h at room temperature. Protein bands were visualized using the enhanced chemiluminescence detection system.

### RNA isolation

Total RNA was extracted from A375 cells that are treated with methanolic leaf extracts of AN or vehicle (0.1 % DMSO) for 24 h using Trizol Reagent (Invitrogen, Carlsbad, CA, USA) according to manufacturer’s protocol. RNA samples were properly dissolved by incubating at 55 °C for 10 min. DNA contamination was removed by treatment with DNase I (Invitrogen #18047019) for 30 min at 37 °C followed by heat inactivation of DNase I prior to quantification using Nano-Drop spectrophotometer. The purified RNA was stored at −80 °C until further use.

### cDNA synthesis and real-time qRT-PCR

First strand cDNA synthesis was done using iScript™ cDNA synthesis kit (Biorad #170-8897) by following manufacturer’s protocols. The cDNA obtained was used to perform real-time qRT-PCR in a Bio-Rad iCycler iQ5TM Real-Time PCR detection system using 2X iQ SYBR Super mix (Invitrogen #K0242). The sequences of primers (from 5′ end to 3′ end) used for qRT-PCR are followed. CyclinD1-F′-TCCAGAGTGATCAAGTGTGA-3′, CyclinD1-R-GATGTCCACGTCCCGCACGT, CDK1-F-CCTTGCCAGAGCTTTTGGAATACC-3′,CDK1-R-GACATGGGATGCTAGGCTTCCTGG, cyclinB-F-TGGAAAAGTTGGCTCCAAAG, cyclin B-R-TCAGAAAAAGCTTGGCAGAGA, Actin-F-GTGGGCATGGGTCAGAAG, Actin-R-TCCATCACGATGCCAGTG. The gene expression levels were determined using the cycle threshold (*C*_t_) method. The mean *C*_t_ values from duplicate measurements were used to calculate the expression of the target gene with normalization to a housekeeping gene and the difference in fold expression was calculated using ΔΔ*C*_t_ method.

### Transient transfection and luciferase assays

To examine the effect of leaf extracts of AN on NF-κB reporter gene activation, B16F10 melanoma cells were seeded in triplicates in 24-well plates (25,000 cells/well). After overnight incubation, cells were transiently transfected with 0.5 μg of pNFκB-Luc-Reporter, and 0.1 μg of pRL-TK (for normalization of transfection efficiency) vectors using fugene 6 transfection reagent. After 6 h, fresh medium was added to cells. 24 h after transfection, cells were treated with different concentrations of methanolic leaf extracts of AN for 48 h followed by TNF-α (10 ng/ml) treatment. The cell culture medium was harvested after 24 h and luciferase activity was measured using dual luciferase assay system according to the manufacturers protocol (Clontech, USA). For renilla luciferase activities, cells were lysed in passive lysis buffer and measured the luciferase activities with luminometer (Promega, Madison, WI, USA). NF-κB-luciferase activities were normalized with that of renilla luciferase values.

### Immunofluorescence

To examine the effect of AN on p65, B16F10 cells were seeded in chamber plates and after overnight incubation cells were treated with methanolic leaf extracts of AN for 48 h. Cells were then stimulated with TNFα (10 ng/ml) for 30 min, and fixed with 4 % paraformaldehyde for 10 min at room temperature followed by permeabilization with 0.2 % Triton X-100. After blocking with 5 % goat serum for 1 h, cells were incubated with p65 primary antibody for overnight at 4 °C. After three washes with phosphate buffered saline (PBS), cells were incubated with anti-FITC conjugated rabbit secondary antibody for 1 h at room temperature. Cells were washed thrice with PBS and cover slips were mounted with VECTA SHIELD mounting medium and fluorescence was captured under fluorescence microscope. The DNA dye 4′,6-diamidino-2-phenylindole (DAPI) was used to visualize the nucleus.

### Statistical analysis

All data were expressed as mean ± standard error (SE) obtained from at least three independent experiments. SPSS software for Windows, version 20 (SPSS, Chicago, IL, USA) was used for statistical analysis. Statistical comparisons among two groups were carried out by student’s *t* test. Differences were considered to be statistically significant at a *P* value of <0.05.

## Results

### Antiproliferative effects of AN on skin cancer cell lines

**Fig. 1:**
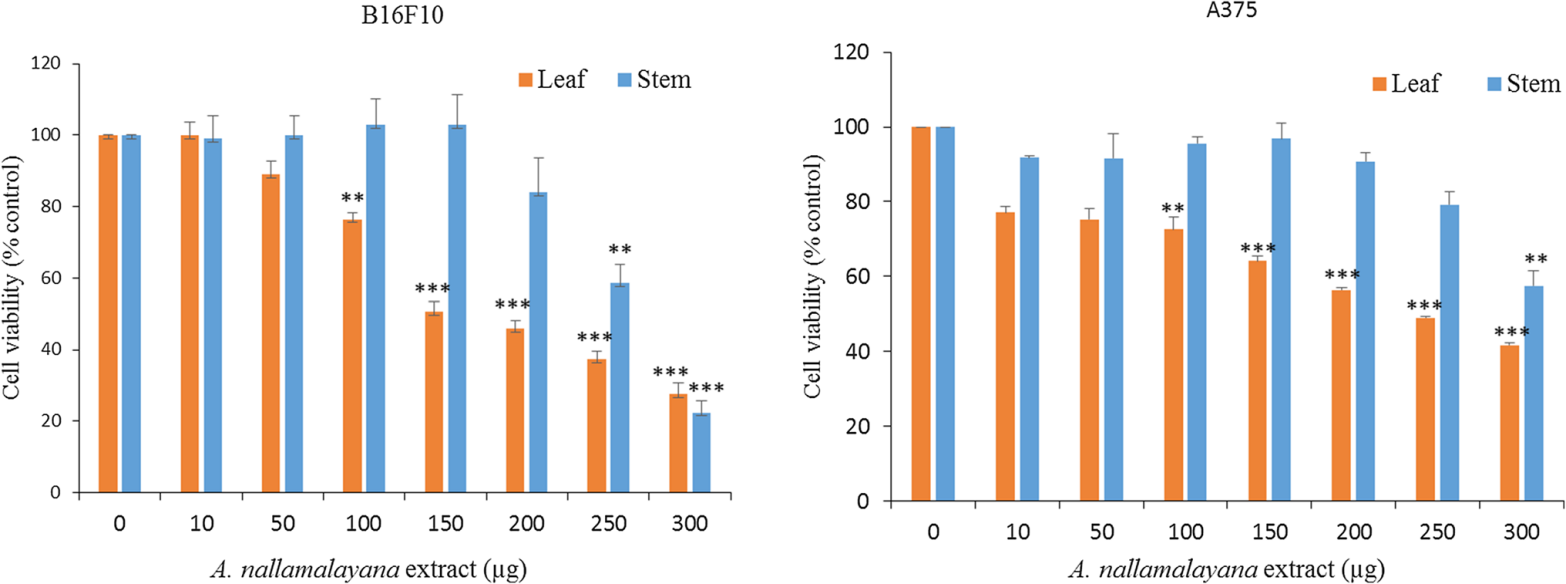
Effect of *A. nallamalayana* on proliferation of A375 and B16F10 melanoma cells. B16F10 cancer cells were seeded in 96 well plates and treated with vehicle (0.1 % DMSO) or indicated concentrations of methanolic extracts from leaf and stem of *A. nallamalayana* for 72 h and subjected to MTT assay. All data presented are the mean ± SE and are representative of three independent experiments. **p < 0.01, ***p < *0.001*, *t* test. ‘0’ denotes the vehicle-treated control cells

**Fig. 2:**
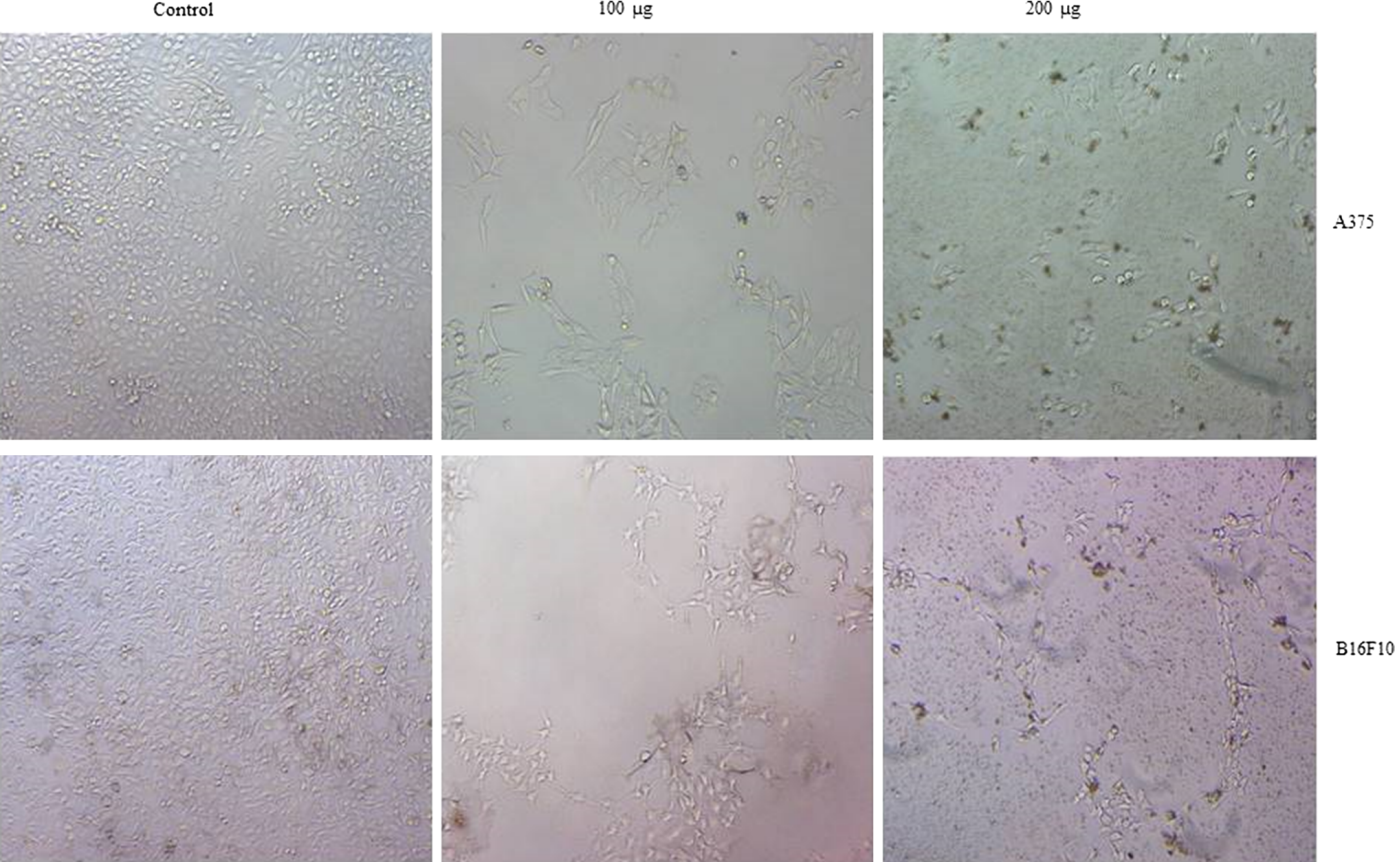
Effect of *A. nallamalayana* on cell number and morphology of A375 and B16F10 skin cancer cells. A375 and B16F10 cells were treated with vehicle (0.1 % DMSO) or indicated concentrations of methanolic leaf extracts of *A. nallamalayana* for 72 h and images were captured under bright filed microscope

Previous reports suggest that *A. paniculata* exhibited antiproliferative effect on various cancer cell lines; however, much is not known about the other species including AN. To know whether AN possesses antiproliferative properties on cancer cell lines, first methanolic extracts of stem and leaf of the test plants were prepared. Then, the extracts were used to test their effect on cell viability using MTT assays. Human skin cancer cell line A375, and mouse skin cancer cell line B16F10 were treated with various concentrations of AN for 72 h. Results showed that methanolic extracts of stem and leaf of AN significantly reduced the cell viability of A375 and B16F10 (Fig. 1) cancer cell lines in dose-dependent manner. When compared to stem extracts, leaf extracts were highly efficient in reducing the cell viability of cancer cell lines. Next, cellular morphology of A375 and B16F10 cells upon leaf extracts treatment were examined. Cell lines were treated with 100 and 200 µg of AN leaf extracts for 72 h and images were captured under bright filed microscope. As shown in Fig. 2, AN extracts reduced the cell number and cells become round and displayed the apoptotic like features.

### AN leaf extracts reduces the colony formation of A375 and B16F10 skin cancer cell lines

**Fig. 3:**
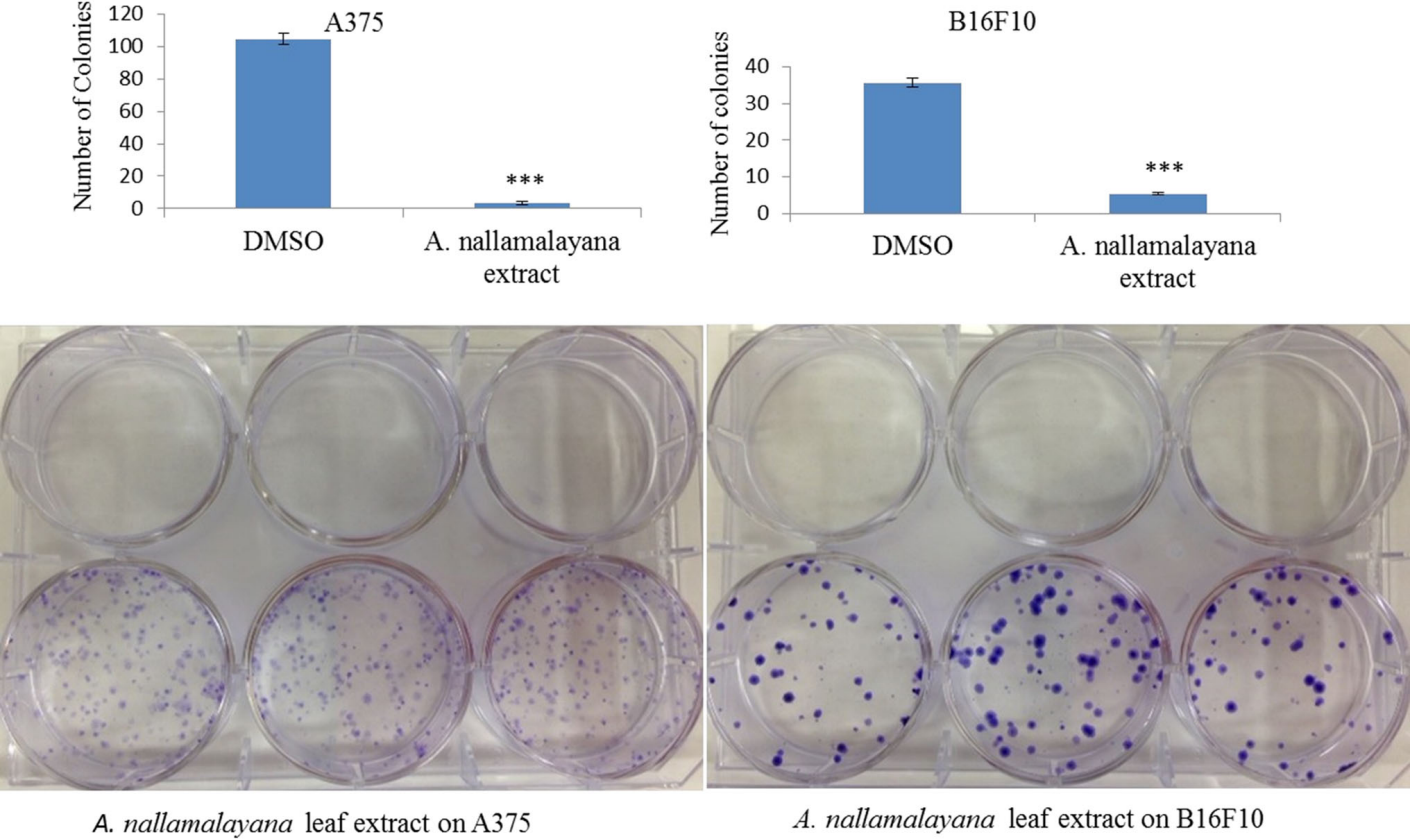
Effect of *A. nallamalayana* on colony formation of A375 and B16F10 cancer cells. 1 × 10^3^ A375 and B16F10 cells were plated in 6 well culture dishes in triplicates and after 24 h cells were treated with vehicle (0.1 % DMSO) or indicated concentrations of *A. nallamalayana* for 48 h. After 7 days colonies were stained with methylene blue and colonies that contain ≥50 cells were counted. All data presented are the mean ± SE and are representative of three independent experiments. ***p < 0.001, *t* test

In addition to reducing the cell viability, other important feature of chemotherapeutic drugs is reducing the survival of cancer cell lines. In the present study, the effect of AN extracts on cell survival was evaluated by employing clonogenic survival assays. Cells were treated with methanolic extracts for 72 h and allowed in growth medium to grow into colonies for 7 days. Cells treated with AN leaf extracts have not shown any colony formations whereas vehicle treated cells have shown well-developed colonies. Untreated A375 and B16F10 cells attain around 80 % of cloning efficiency, whereas AN treatment significantly reduced the cloning efficiency of skin cancer cells (Fig. 3). These results demonstrated that AN reduces the survival of skin cancer cell lines.

### AN leaf extracts induce the apoptosis of A375 and B16F10 cell lines

**Fig. 4:**
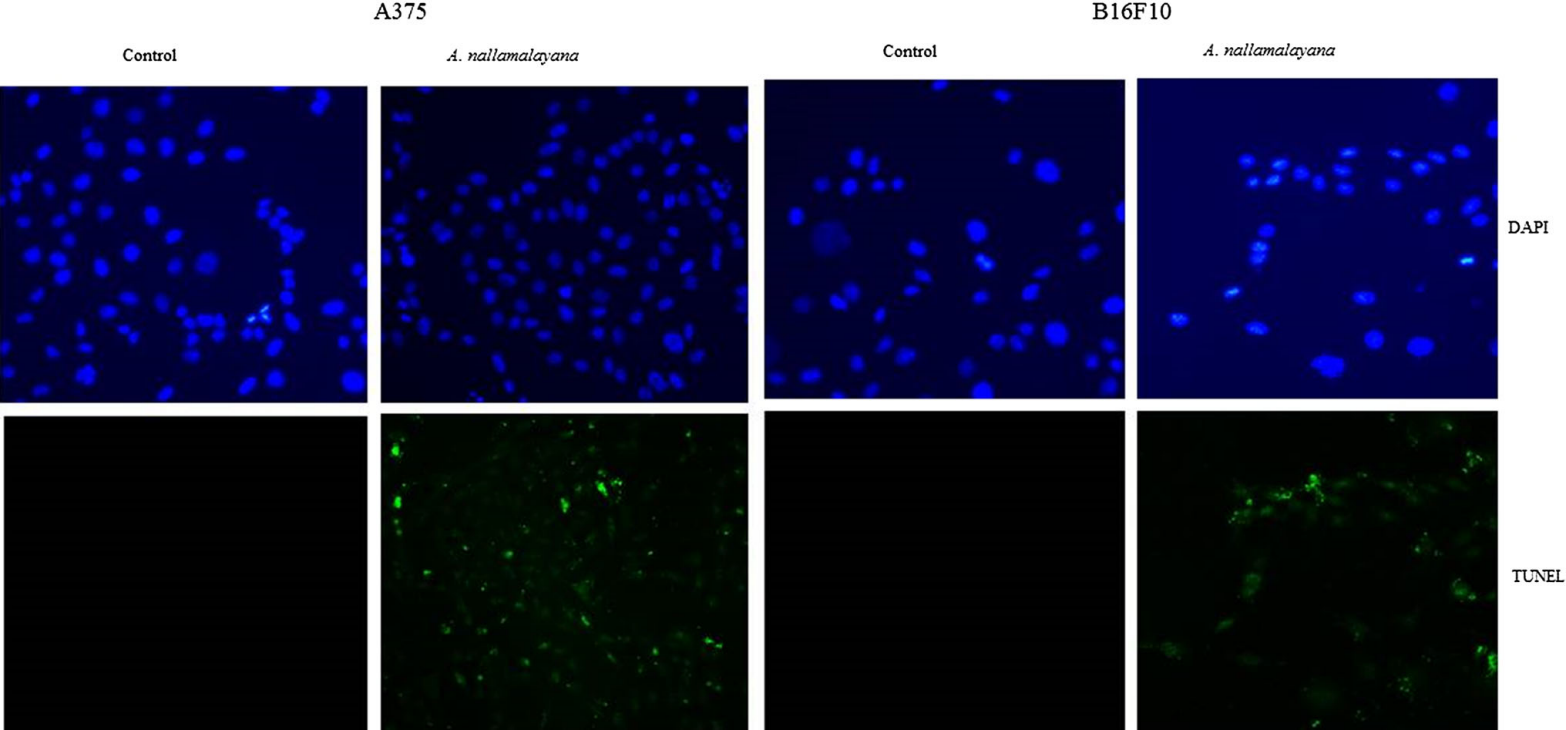
Effect of *A. nallamalayana* on apoptosis of A375 and B16F10cancer cells. A375 and B16F10 cells were seeded in chamber plates and treated with vehicle (0.1 % DMSO) or 200 µg *A. nallamalayana* for 72 h. Then cells were subjected to TUNEL apoptosis assay according to manufacturer’s protocol. DAPI was used to identify the nucleus and data are representative of three independent experiments

Treatment with AN resulted in rounding of the cells and exhibited apoptotic-like features. To further determine whether AN extracts induces apoptosis of skin cancer cells, the extent of DNA fragmentation was determined by terminal deoxynucleotidyl transferase-mediated dUTP nick end labeling (TUNEL) assay and the apoptotic cells were marked with higher fluorescein isothiocyanate (FITC) fluorescent intensity with green color under a microscope. To localize the nucleus, DAPI was used as counter stain. As shown in Fig. 4, AN treatment significantly induces the apoptosis of B16F10 and A375 cell lines, respectively.

### AN leaf extracts alters the expression of proteins involved in G2/M phase transition

**Fig. 5:**
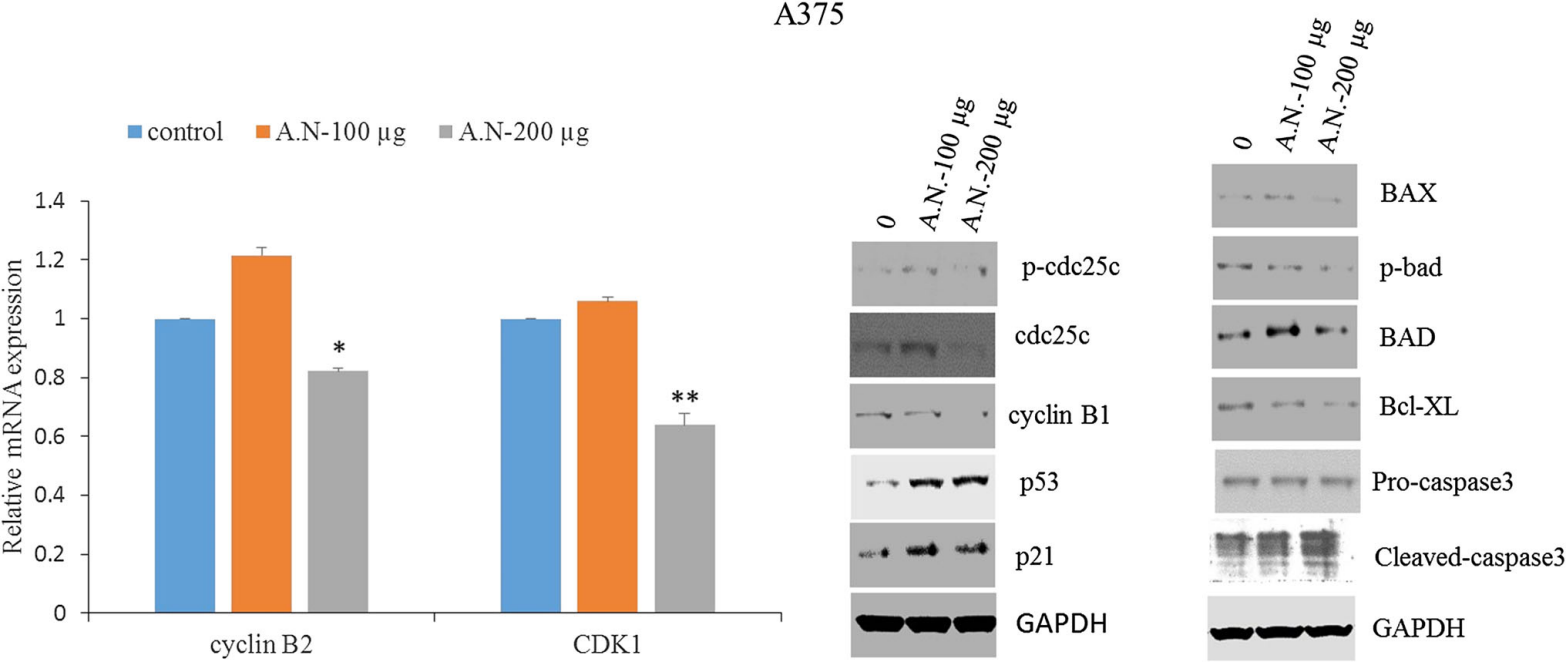
**a** Effect of *A. nallamalayana* (A.N) on expression of cyclin B1 and cdk1 in A375 cancer cells. A375 cells were treated with vehicle (0.1 % DMSO) or indicated concentrations of *A. nallamalayana* for 24 h. RNA was isolated and subjected to RT-qPCR using the primers specific for cyclin B1 and cdk1. All data presented are the mean ± SE. *p < 0.05, **p < 0.01, *t* test. **b** Effect of *A. nallamalayana* (A.N) on protein levels of G2/M phase proteins. Cell lysates of A375 skin cancer cells treated with indicated concentrations of *A. nallamalayana* for 24 h and whole cell lysates were subjected to western blot analysis with p-cdc25c, cdc25c, cyclin B1, p53 and p21 antibodies. GAPDH used as an internal control. **c** Effect of *A. nallamalayana* (A.N) on protein levels of proapoptotic and antiapoptotic proteins. Cell lysates of A375 skin cancer cells treated with indicated concentrations of *A. nallamalayana* for 48 h and whole cell lysates were subjected to western blot analysis with BAX, p-BAD, BAD, Bcl-XL, procaspase-3 and cleaved caspase-3 antibodies. GAPDH used as an internal control

To investigate the molecular mechanisms involved in the G2/M arrest A375 cell lines were treated with various doses of AN for 24 h and tested the expression and phosphorylation of several proteins that are involved in the G2/M transition using western blotting and qRT-PCR. After treating the cells with indicated concentrations of AN, the levels of cyclin B1 were analyzed by western blotting and real-time PCR. As shown in Fig. 5a, expression levels of cyclin B1 mRNA as well as protein (Fig. 5b) were down-regulated in a concentration-dependent manner. However, the alterations in the expression of cyclins were not sufficient to explain the G2/M arrest induced by AN. G2/M progression depends not only upon the expression of Cdk inhibitors but also on phosphorylation of Cdk1 (Cdc2), the indicative of Cdc2 activity, which is negatively regulated by the phosphorylation of Thr14 and Tyr15. Cdc25c is a protein phosphatase that activates Cdc2 by dephosphorylation. Cdc25c is phosphorylated at ser216 in response to DNA damage at G2/M checkpoint and interacts with members of the 14-3-3 family of proteins, leading to its sequestration in the cytoplasm and thereby preventing premature mitosis. We next assessed the effect of AN on Cdc25c phosphorylation status. Western blot analysis revealed that AN treatment increased the levels of phosphorylated Cdc25c (Fig. 5b). We also examined the effects of AN on Cdc25c protein expression, and observed that Cdc25c was significantly downregulated in AN-treated A375 cells (Fig. 5b). The cyclin-dependent kinase 1 (Cdk1), formerly called Cdc2, interacts with cyclin B1 and forms Cdk1/cyclin B1 complex which regulates the progression from G2- to M phase. The effect of AN extracts on Cdk1 expression by qRT-PCR analysis was examined. As shown in Fig. 5a, AN treatment significantly reduces the expression of cdk1 dose dependently in A375 cells.

To further examine whether AN induces CDK inhibitor proteins, p21 protein levels were measured using western blot analysis up on AN extract treatments on A375 cells. AN treatment also induced a significant increase in p21 protein expression dose dependently (Fig. 5b). The expression of p21 is regulated by either a p53-dependent or p53-independent mechanism. In addition, p53 induces the cell cycle arrest as well apoptosis by regulating the expression of its target genes p21 and PUMA. To know whether the growth-inhibitory effect of AN was dependent on p53 status, p53 protein levels were tested following treatment. As shown in Fig. 5b, the expression of p53 was significantly increased in A375 cells following AN treatment.

### AN leaf extracts enhances the expression of apoptotic proteins in A375 cells

To better understand how AN extract induces apoptosis and reduced the cell survival of skin cancer cell lines; the expression of Bcl-2 family proteins which involved in apoptosis and cell survival was analyzed. Pro-apoptotic proteins, including BAD and BAX, can trigger the apoptotic cascade by forming pores in the mitochondrial membrane. The expression of pro-apoptotic molecules such as BAX, BAD and anti-apoptotic molecule Bcl-XL following AN treatment was measured. As shown in Fig. 5c, western blot analysis demonstrated that the expression of BAD, a pro-apoptotic BH3-only member of the Bcl-2 family was significantly increased in A375 cells following AN treatment compared to control cells (Fig. 5c). The levels of p-BAD was significantly reduced and BAD protein levels were significantly increased dose dependently following AN extract treatments in A375 cells. Further the protein levels of anti-apoptotic molecule Bcl-XL was examined following AN extract treatment. As shown in Fig. 5c, western blot analysis showed the significant down-regulation of Bcl-XL levels in AN-treated A375 cells compared to controls. The results suggest that regulatory proteins are involved in AN leaf extract induced apoptosis of A375 cells.

Activation of pro-apoptotic proteins, such as BAD and BAX, results in induction of apoptotic cascade by forming pores in the mitochondrial membrane and subsequent increase in cytosolic concentration of cytochrome-*c*, which in turn activates effector caspases such as caspase-3. The whole cell lysates were subjected to western blotting to recognize the cleavage of caspase-3 in A375 cells. As shown in the Fig. 5c, AN extract treatment significantly increased the levels of cleaved caspase-3 in a dose-dependent manner, suggests that AN leaf extract induces apoptosis in A375 cells.

### AN leaf extracts inhibits the nuclear translocation

**Fig. 6:**
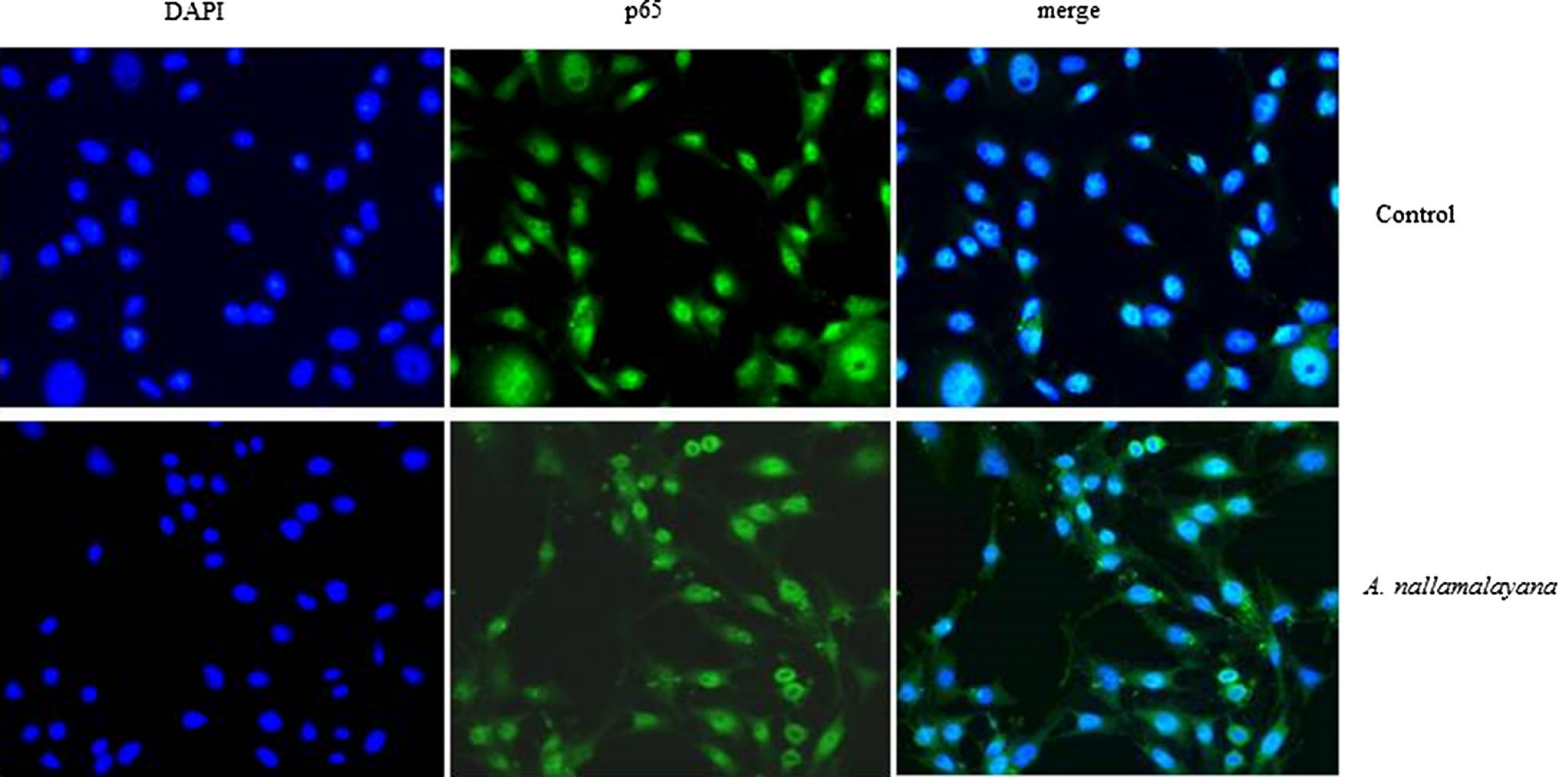
Effect of *A. nallamalayana* (A.N) on TNFα induced nuclear translocation of p65. B16F10 cells were seeded on to coverslips in 24 well plates and pretreated with vehicle or *A. nallamalayana* (A.N) leaf extracts for 48 h, followed by stimulation with TNFα (10 ng/ml) for 30 min. Cells were then fixed in 4 % paraformaldehyde and incubated with p65 primary antibody followed by FITC conjugated secondary antibodies for 1 h at room temperature. Fluorescence was captured under fluorescence microscope. DAPI was used to visualize the nuclei. Upon stimulation with TNFα, P65 was translocated to nucleus. *A. nallamalayana* (A.N) treatment resulted in the inhibition of nuclear translocation of p65 following TNFα stimulation and most of the p65 is localized in the cytosol

To further examine the mechanism of inhibition of cell proliferation following AN extract treatment, the activity of NF-κB signaling pathway which is known to play crucial roles in inflammation, cell survival and apoptosis of skin cancer cell lines were assessed. Briefly NF-kB nuclear translocation was induced with TNFα in B16F10 cells, followed by AN leaf extract treatment for 24 h and nuclear levels of p65 was measured using immunofluorescence. The nuclear translocation of p65 was increased following TNFα stimulation in B16F10 cells, interestingly AN extract treated cells significantly reduced the TNFα-induced nuclear p65 levels in B16F10 cells (Fig. 6) and p65 was mostly localized in cytosol.

### AN leaf extracts reduces reporter gene activation of NF-κB And the expression of NF-κB target genes

**Fig. 7:**
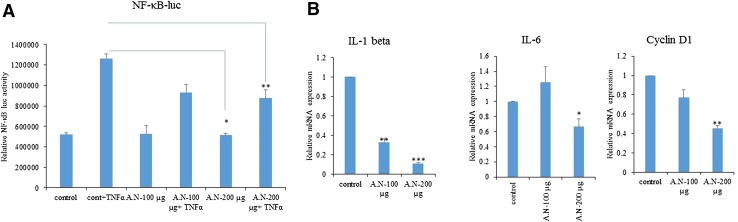
Effect of *A. nallamalayana* (A.N) on TNFα induced NF-kB reporter gene activation and its target genes. A. B16F10 cells were seeded in 24-well plates and transiently transfected with pNF-κB-Luc-Reporter and pRL-TK (for normalization of transfection efficiency) plasmids using fugene 6 transfection reagent as described in materials and methods. After 6 h cells were treated with indicated concentrations of *A. nallamalayana* (A.N) for 48 h followed by TNFα stimulation for 24 h. Cells were lysed in passive lysis buffer and luciferase values were measured using dual luciferase assay system. The firefly luciferase activities were normalized against renilla luciferase activities. *A. nallamalayana* (A.N) reduced the NF-kB-dependent reporter activity in a dose-dependent manner. **b** A375 cells were treated with vehicle (0.1 % DMSO) or indicated concentrations of *A. nallamalayana* (A.N) for 24 h. RNA was isolated and subjected to RT-qPCR using the primers specific for IL-1 beta, IL-6 and cyclin D1. All data presented are the mean ± SEM. *p < 0.05, **p < 0.01, ***p < 0.001 *t* test

Once NF-κB is activated, it further translocates to nucleus and activates expression of its target genes. Study was extended to know the effect of AN extract treatment on NF-κB activation. B16F10 cells that were transiently transfected with pNF-κB-Luc-Reporter plasmid, and pRL-TK (for normalization of transfection efficiency) plasmid, were treated with AN leaf extracts followed by TNFα stimulation. As shown in Fig. 7a, NF-κB reporter gene activity is increased significantly following TNF-α stimulation compared to unstimulated B16F10 cells and this enhanced reporter activity is potentially inhibited by AN leaf extract. Results clearly suggest that AN extracts significantly inhibit NF-κB signaling pathway activation that leads to reduction in cell proliferation. NF-κB enhances the cell proliferation, survival and activates the inflammation by increasing the expression of IL-1 beta, IL-6 and cyclin D1 which are well known NF-κB-regulated genes. Gene expression levels of IL-1 beta, IL-6 and cyclin D1 were tested using qRT-PCR. As shown in Fig. 7b, the expression levels of IL-1 beta, IL-6 and cyclin D1 were decreased dose dependently after AN extract treatment in A375 cells. Results suggest that AN treatment regulates the expression of NF-κB target gene expression by inhibiting NF-κB activation.

## Discussion

Cancer is one of the leading causes of death worldwide and the numbers of cancer cases are gradually increasing globally. There are different kinds of medicines available in the market to treat the several types of cancer but till date no drug is found to be safe and effective. One of the major impediments in the cancer chemotherapy is the toxicity of the established chemotherapeutic drugs. In recent years, the use of plants and plant derived products has proved to be effective and safe in the treatment and management of several types of cancers. Currently, most of the research work on cancer drugs is based on plants and plants-derived natural products. Since ancient time, plant products were used for health benefits by all cultures as well as source of medicines (Ignacimuthu et al. 2006; Elujoba et al. 2005; Tomlinson and Akerele 1998). Several natural products and their analogs have been identified as potent anti-cancer agents and the identification of anti-cancer property of various plants is being unraveled in significant manner. In the present study we have chosen a beautiful herb, endemic to few pockets of Nallamala hills in Indian peninsular, known for treating various ailments such as mouth ulcers and leukoderma in folklore medicine, persisting with antimicrobial and anti-psoriatic properties. Essentially, it is a rare plant with flourishing medicinal properties.

In the study, attempts were made to examine whether AN persists with any anti-cancerous properties such as anti-proliferative, anti-inflammatory and pro-apoptotic activities on A375 and B16F10 skin cancer cell lines. MTT cell viability assays and clonogenic assays revealed that AN leaf extracts have significant anti-proliferative effects on skin cancer cell lines. Further TUENL assays also demonstrated that AN induced the apoptosis of skin cancer cell lines. These results supports the notion that AN leaf extracts reduce the cell viability, and concomitantly activate the apoptosis.

Cell cycle checkpoints are important regulatory mechanisms that ensure the proper passage of cells through the cell cycle. The G2/M and G1/S checkpoints are critical in maintaining DNA integrity and essential in executing the proper cell cycle events. The G2/M checkpoint is one of the important check points that blocks the entry into mitosis when DNA is damaged (Taylor and Stark 2001). It was known that loss of regulation of these checkpoints resulted into transformation and progression of cancer cells. A protein kinase complex consisting of a catalytic subunit, Cdk1 (previously known as cdc2) and the cyclin B protein (Cdk1/cyclin B1 complex) regulates the central and rate-limiting function in the transition from G2 to M phase (Norbury 1992). In response to DNA damage, the cdc2/cyclin B1 complex causes a delay in cell cycle progression at G2/M phase to allow DNA repair before cells enter mitosis. This was achieved by phosphorylating cytoskeleton proteins such as lamins and histone H1 (Johnson and Walker 1999). Cdk1/cyclin B1 complex formation itself is not enough for cell cycle progression. However, the event of dephosphorylation of cdk1 at the Tyr-15 site through cdc25c phosphatase is required for the optimal activation of the cdk1/cyclin B1 complex (Sampath and Plunkett 2001). In the present study, AN treatment of A375 skin cancer cells resulted in inhibition of various cell cycle proteins. To elucidate the molecular mechanism, alterations in the levels of various cell cycle proteins were estimated and found that a decrease in levels of Cdk1, cyclin B1 and cdc25c following AN extract treatment, suggests the inhibitory activities of AN leaf extracts on G2/M phase of cell cycle arrest.

The transcriptional factor p53 functions as a guardian of the genome by modulating appropriate responses to stress as well as DNA damage. In addition, several studies showed the significant role of p53 in cell cycle checkpoint control. It has been showed that p53 blocks cell cycle at G2/M phase through direct inhibition of cdc2 kinase (Taylor and Stark 2001). It was also known that p53 regulate the G2/M phase transition through the induction of p21, a protein that normally inhibits cyclin B1-Cdc2 complexes in the cytoplasm (Bunz et al. 1998) or through the induction of apoptosis (Shaw et al. 1992). The present study revealed that AN leaf extract treatment resulted in stabilization of p53 protein expression. Further, it was found that several apoptotic genes including BAX, BAD were upregulated and survival genes such as Bcl-XL was down regulated in A375 skin cancer cell lines. Moreover, AN significantly increased the cleavage of Caspase-3. p21 is an inhibitor of cyclin/cyclin-dependent kinase complexes and its induction is mediated by both p53-dependent and p53-independent mechanisms and plays essential roles in both G1 and G2 cell cycle arrest in response to DNA damage and cell senescence (Roninson 2002). The study demonstrates that AN treatment significantly increased the expression of cdk inhibitor, p21 which contributed to the G2/M arrest and growth inhibition of A375 skin cancer cells.

Solar UV radiation-induced skin cancer or photocarcinogenesis is a complex process that involves a cascade of individual steps which includes apoptosis, proliferation, autophagy, DNA repair, checkpoint signaling, metabolism and inflammation. Several signaling pathways regulate the events. Of which NF-κB signaling pathway plays key roles in inflammation, cellular proliferation, and induction of cancers (Kim and He 2014). NF-κB is a family of dimeric transcription factors that play vital roles in diverse physiological processes and various human malignancies. NF-κB family includes RelA/p65, RelB, c-Rel, p50/p105 and p52/p100. In unstimulated cells, NF-κB/Rel dimers are bound to IκB and retained in cytoplasm. In response to stimulus such as TNFα, LPS the IκB proteins are phosphorylated by an activated IκB kinase (IKK) complex at positions 32 and 36 followed by polyubiquitination and degradation by 26S proteasome releasing free NF-κB dimers. Then the p50/65 complex translocates to nucleus and binds to their target gene promoters and drives their transcription (Karin et al. 2002; Nishikori 2005). NF-κB is activated by UVB radiation and contributes to UVB-induced skin carcinogenesis (Syed et al. 2012). Because inhibition of NF-κB activation has been linked with anti-tumor activities, it was hypothesized that AN mediates their effect at least partly through inhibition of NF-κB activation. In this study, the inhibitory effects of AN extracts on nuclear translocation of p65 were examined. Results showed that TNFα-induced nuclear translocation and NF-κB-dependent reporter gene expression were inhibited following AN treatment. Moreover, AN extracts reduced the NF-kB reporter gene activation and also reduced the NF-kB targeted genes expression including IL-1 beta, IL-6 and cyclin D1.

In conclusion, methanolic leaf extracts of the endemic herb AN reduced the proliferation and induced apoptosis of skin cancer cell lines. Mechanistic studies revealed that AN targets the G2/M phase by altering the expression of proteins involved in G2/M phase, by inducing expression of pro-apoptotic proteins and by inhibiting the NF-kB pathway activation. The findings might help in providing the rationale to initiating in vivo studies to examine the efficacy of AN as chemopreventive agent against skin cancer.
